# Development and Validation of a Gene Signature for Prediction of Relapse in Stage I Testicular Germ Cell Tumors

**DOI:** 10.3389/fonc.2020.01147

**Published:** 2020-07-30

**Authors:** Jian-Guo Zhou, Jie Yang, Su-Han Jin, Siyu Xiao, Lei Shi, Ting-You Zhang, Hu Ma, Udo S. Gaipl

**Affiliations:** ^1^Department of Oncology, The Second Affiliated Hospital of Zunyi Medical University, Zunyi, China; ^2^Department of Radiation Oncology, Universitätsklinikum Erlangen, Erlangen, Germany; ^3^Comprehensive Cancer Center Erlangen-EMN, Erlangen, Germany; ^4^Guangdong Lung Cancer Institute, Guangdong Provincial People's Hospital, Guangdong Academy of Medical Sciences, School of Medicine, South China University of Technology, Guangzhou, China; ^5^Department of Orthodontics, Affiliated Stomatological Hospital of Zunyi Medical University, Zunyi, China; ^6^Department of Clinical Laboratory, The Sixth Affiliated Hospital of Sun Yat-sen University, Guangzhou, China

**Keywords:** testicular germ cell tumors, The Cancer Genome Atlas, risk score model, relapse, GEO

## Abstract

**Background:** Testicular germ cell tumors (TGCTs) are commonly diagnosed tumors in young men. However, a satisfactory approach to predict relapse of stage I TGCTs is still lacking. Therefore, this study aimed to develop a robust risk score model for stage I TGCTs.

**Method:** RNA-sequence data of stage I TGCTs and normal testis samples were downloaded and analyzed to identify different expression genes. Gene-based prognostic model was constructed in The Cancer Genome Atlas (TCGA) using least absolute shrinkage and selection operator (LASSO) regression analysis and validated in GSE99420 dataset. Potential biological functions of the genes in prognostic model were determined via Gene Set Enrichment Analysis (GSEA) between high-risk and low-risk patients.

**Results:** A total of 9,391 differentially expressed genes and 84 prognosis-related genes were identified. An eight-gene-based risk score model was constructed to divide patients into high or low risk of relapse. The low-risk patients had a significantly better relapse-free survival (RFS) than high-risk patients in both training and validation cohorts (HR = 0.129, 95% CI = 0.059–0.284, *P* < 0.001; HR = 0.277, 95% CI = 0.116–0.661, *P* = 0.004, respectively). The area under the receiver operating characteristic curve (AUC) values at 5 years was 0.805 and 0.724 in the training and validation cohorts, respectively. Functional enrichment analyses showed that DNA replication, ribosome, cell cycle, and TGF-beta signaling pathway may contribute to the relapse process.

**Conclusion:** In summary, our analysis provided a novel eight-gene signature that could predict RFS in stage I TGCT patients.

## Introduction

Testicular germ cell tumors (TGCTs) are the most commonly diagnosed tumors in young men (1–3). Most patients with TGCTs have a good prognosis; stage I patients who receive only surgical resection have an 85.5–95% 5 year survival rate, and even patients with metastasis still have a cure rate of ~80%, because TGCTs are sensitive to radiotherapy and chemotherapy, such as cisplatin and other cytostatic agents (1, 4–6). Previous studies have shown that advanced TNM stage, high serum tumor marker level (7–9), non-seminoma or predominantly embryonal carcinoma histology (10), lymph vascular invasion (11–13), rete testis invasion (14, 15), and large tumor size (15, 16) are risk factors of poor prognosis or relapse. However, based on conventional clinical or pathological characteristics, the prediction of recurrence is still not sufficiently accurate and remains controversial in TGCTs (1, 15), especially in stage I patients who are subjected to surveillance after radical orchiectomy (5). The lack of ideal prognostic biomarkers makes individualized therapy difficult. Therefore, the development of a novel discriminatory signature to identify the high-risk relapse subset of stage I TGCTs is urgently required.

The Cancer Genome Atlas (TCGA) database provides large-scale samples of TGCTs with abundant gene expression profiles and clinical information that enable a comprehensive analysis of TGCTs. In addition, another public database, the Genotype-Tissue Expression (GTEx) Project, contains the expression data of normal tissue (17, 18) that enable genomics analysis between TGCTs and normal testis.

By analyzing the clinical and expression data from TCGA and GTEx, we aimed to develop a risk score model based on gene expression in TGCTs and to explore the underlying mechanism. The results from this study may provide a new tool to guide treatment decisions for TGCT patients.

## Methods

### Overall Workflow and Data Downloading

**Figure 1:**
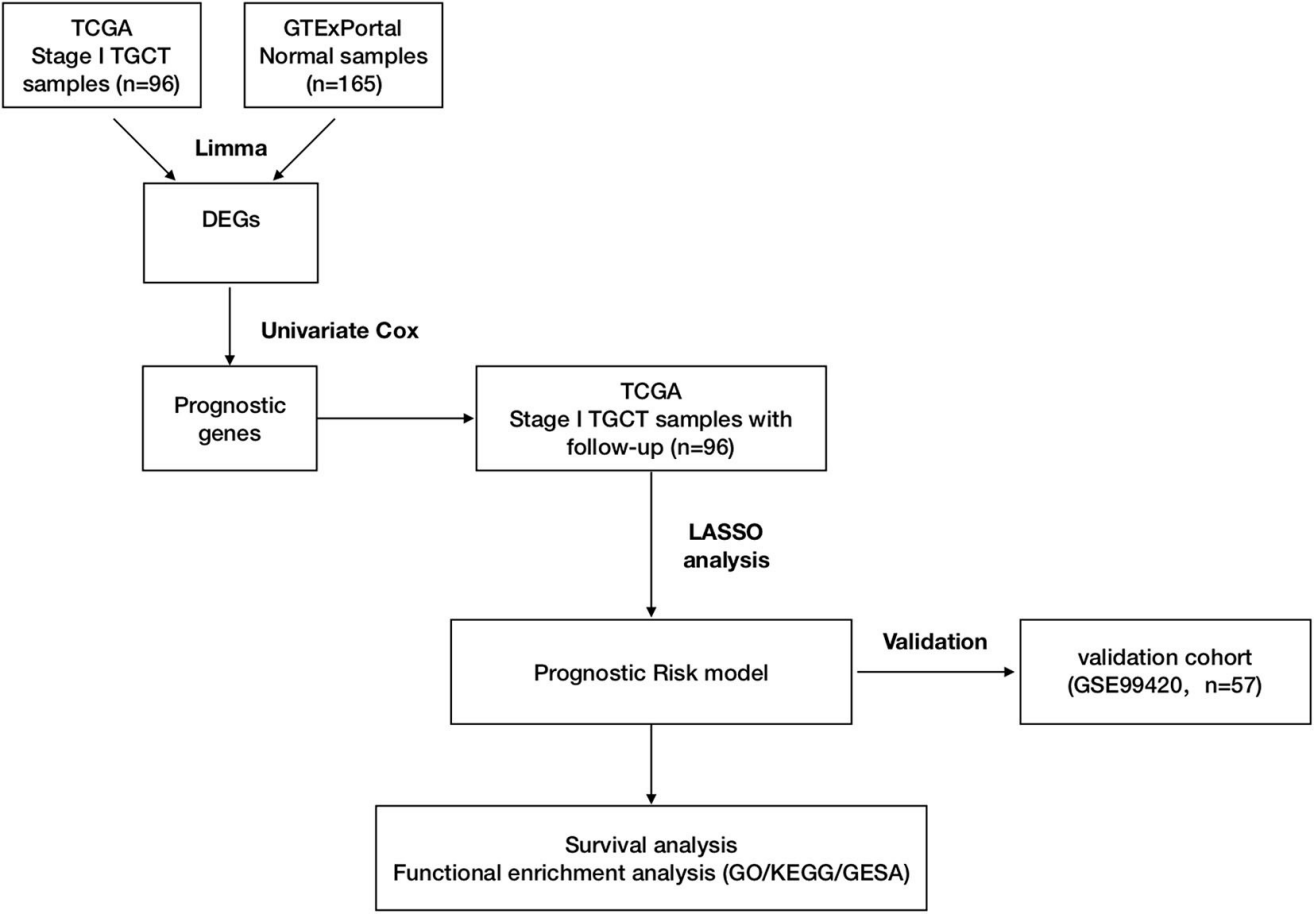
Workflow of construction and validation of the risk score model. The figure shows the overall workflow of the prognostic model construction and validation.

Gene expression data for TGCTs, clinical information, and mutation data were downloaded from TCGA data portal (dated to February 18, 2019) by TCGAbiolinks (version 2.14.0) (19). Data of follow-up information were also downloaded from TCGA Pan-Cancer Clinical Data Resource (TCGA-CDR) to ensure data consistency from TCGA data portal (20). Human normal testis tissue gene expression profiles were downloaded from the GTEx Portal as previously described (17, 21, 22). Raw read counts were used for differential expression analysis. Transcripts per kilobase million (TPM) were used for survival analysis and model development. The GSE99420 dataset was downloaded from the Gene Expression Omnibus (GEO), which contains 60 stage I TGCTs from Princess Margaret Cancer Centre, Toronto. This dataset was based on GPL14951 platform (Illumina HumanHT-12 WG-DASL V4.0 R2 expression beadchip). Follow-up information was obtained from the contributor of GSE99420. Stage I TGCT samples in TCGA-TGCT cohort were set as training cohort for prognostic risk model construction and GSE99420 cohort as validation cohort. Figure 1 shows the overall design of this study.

### Differentially Expressed Genes and Prognostic Gene Selection

Identification of differentially expressed genes (DEGs) were using the Limma package in R (23). Genes with more than 2-fold expression were regarded as differentially expressed (adjust *P* < 0.05). All gene expression data from different datasets were separately log2 transformed and normalized by “limma” package in R to eliminate biological deviation. Then the genes, which were correlated with relapse-free survival (RFS), analyzed by univariate Cox proportional hazards regression (s*urvival*, version 2.43-3), were identified as prognostic genes (*P* < 0.05) for the subsequent construction of the prediction model (24, 25).

### Risk Score Model Construction and Validation

Previously selected genes were further screened and confirmed by least absolute shrinkage and selection operator (LASSO) regression using the R package (*glmnet*, version 3.0-2) (26). The prognostic risk score was calculated with the following formula: risk score = β_1_^*^ (expression of RNA_1_) + β_2_^*^ (expression of RNA_2_) + ··· + β_n_^*^ (expression of RNA_n_). We randomly repeated the procedure 200,000 times to construct the best risk score model by LASSO regression. Risk scores for each patient were calculated using the formula. Time-dependent receiver operating characteristic (ROC) curves were constructed to evaluate the optimal cutoff for risk scores in the training and validation cohort using the R package (*survivalROC*, version 1.0.3) (27). On the basis of their cut-off, patients were classified into either the high-risk group or low-risk group, and then survival analysis was compared between high- and low-risk patients to test the predictive power in the validation cohort.

### Functional Enrichment Analysis

DEGs between high- and low-risk patients were considered hub genes for relapse in TGCT patients. Functional enrichment analyses and Gene Set Enrichment Analysis (GSEA)-based (28) Kyoto Encyclopedia of Genes and Genomes (KEGG) and Gene Ontology (GO) analyses were conducted by the R package (*clusterProfiler*, version 3.14.3) (29) to explore different molecular mechanisms and involved pathways between high- and low-risk patients. Normalized enrichment score was acquired using gene set permutations with 1,000 times and the cutoff *P*-value was 0.05 to filter the significant enrichment results.

### Statistical Analysis

All statistical analyses were performed using the software R (version 3.5.2) with corresponding packages. Continuous variables were presented as means ± SD and categorical variables were displayed as percentage. With the use of an R package (*survival*, version 2.43.3) (24, 25), Kaplan–Meier survival analysis and the log-rank test were employed to compare RFS between the high-risk and low-risk groups in the training and validation cohorts. The area under the ROC curve (AUC) was calculated using R package (*survivalROC*) (27) to estimate the prognostic power in two groups. Univariate and multivariate Cox regression analyses were performed between high-risk and low-risk groups in all stage I TGCTs and different subgroups.

## Results

### Baseline Characteristics of Patients

After relapse time of <30 days was excluded, 96 stage I TGCT patients with relapse information were selected from TCGA-TGCT dataset as training cohort. GSE99420 included 57 stage I TGCT patients, which had relapse information, but without detail clinical information. The detailed baseline characteristics of the training and validation cohort are listed in Table 1.

**Table 1:** Baseline characteristics of Stage I TGCT patients in training and validation cohort.

	**TCGA-TGCT (*n =* 96)**	**GSE99420 (*n =* 57)**
Age, Mean (SD)	32.80 (9.85)	34.8 (8.76)
**Race, *n* (%)**		/
Other	9 (9.4)	
White	87 (90.6)	
**Histological type, *n* (%)**		
Seminoma	60 (62.5)	30 (52.63)
Non-seminoma	36 (37.5)	27 (47.37)
**Lymphovascular invasion, *n* (%)**		/
No	62 (64.6)	
Yes	34 (35.4)	
Tumor (T) classification, *n* (%)		/
T1	64 (66.7)	
Other	32 (33.3)	
**Serum markers (S) classification**, ***n*** **(%)**		/
S0	38 (39.6)	
Other	58 (60.4)	
**Postoperative treatment**, ***n*** **(%)**		/
None	61 (63.5)	
Yes	35 (36.5)	
**Relapse status**		
Relapse	29 (30.0)	27 (47.37)
No-relapse	67 (70.0)	30 (52.63)

### Identification of Differentially Expressed Genes

**Figure 2:**
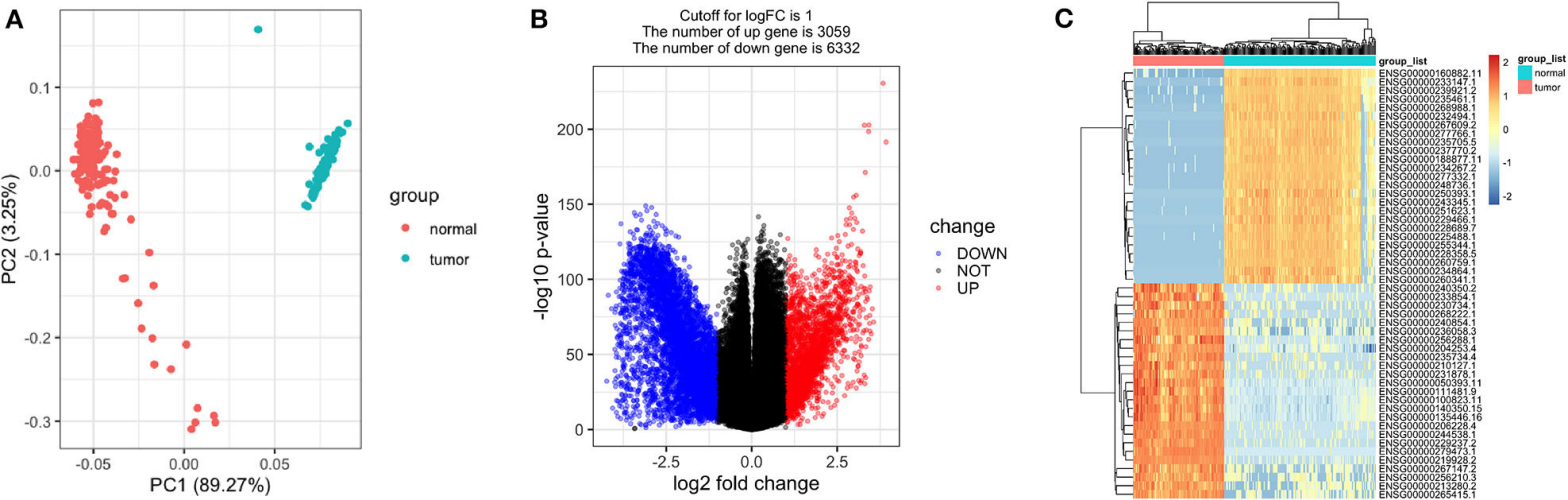
Differentially expressed genes between stage I testicular germ cell tumors (TGCTs) and normal testis tissues. Principal component analysis plot **(A)**, volcano plot **(B)**, and heatmap **(C)** demonstrating differentially expressed genes between stage I TGCT and normal tissues.

As expected, the tumor and normal tissues showed significantly different clusters in principal component analysis (PCA) (Figure 2A). Differential expression analyses identified 9,391 genes differentially expressed between the stage I TGCT samples and testis tissues (3,059 upregulated and 6,332 downregulated genes, Figures 2B,C). The details of differential expression genes are shown in Supplementary Table 1.

### Development and Validation of the Risk Score Model Based on Gene Expression

Among the 9,391 candidate DEGs, 84 were identified as being independently associated with RFS in univariate Cox regression analysis (Supplementary Table 2). Based on the results of LASSO Cox regression analysis, a risk formula was developed. The risk score was calculated as follows: [Expression of GPR174 ^*^ (−1.6762644) + Expression of TCTEX1D1 ^*^ (−1.4554797) + Expression of TMEM89 ^*^ (−1.0862306) + Expression of CST2 ^*^ (−0.8966736) + Expression of SRARP ^*^ (−0.6258739) + Expression level of GSC ^*^ (−0.3162878) + Expression of PLEKHS1 ^*^ (−0.2249954) + Expression of FLG2 ^*^ (1.2350859)]. Table 2 summarized the information of the eights genes.

**Table 2:** Information of the 8 genes in risk score model.

**Ensembl ID**	**Gene Synonyms**	**Chromosomal location**	**Gene type**	**Description**	**LASSO coefficient**
**Protective genes**					
ENSG00000147138	GPR174	Xq21.1	Protein coding	G Protein-coupled receptor 174	−1.6762644
ENSG00000152760	TCTEX1D1	1p31.3	Protein coding	Tctex1 domain-containing protein 1	−1.4554797
ENSG00000183396	TMEM89	3p21.31	Protein coding	Transmembrane protein 89	−1.0862306
ENSG00000170369	CST2	20p11.21	Protein coding	Cystatin SA	−0.8966736
ENSG00000183888	SRARP	1p36.13	Protein coding	Steroid receptor associated and regulated protein	−0.6258739
ENSG00000133937	GSC	14q32.13	Protein coding	Homeobox protein goosecoid	−0.3162878
ENSG00000148735	PLEKHS1	10q25.3	Protein coding	Pleckstrin homology domain-containing family S member 1	−0.2249954
**Risky genes**					
ENSG00000143520	FLG2	1q21.3	Protein coding	Intermediate Filament-Associated And Psoriasis Susceptibility Protein	1.2350859

In this risk score model, seven genes were positively associated with RFS, and one was negatively associated with RFS. Risk scores for each patient were calculated with this formula (Supplementary Table 3).

**Figure 3:**
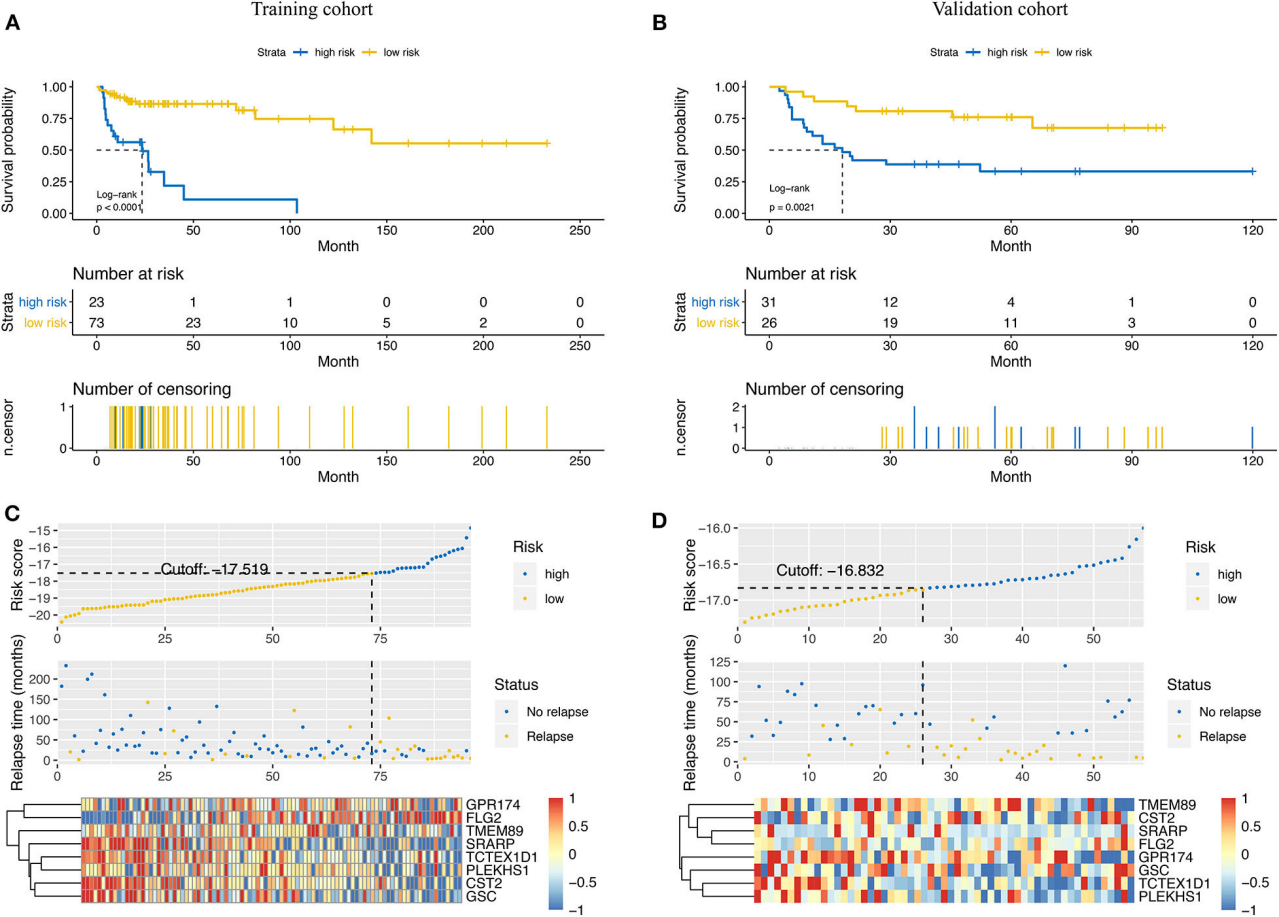
Prediction power of the risk score model in the training cohort and the validation cohort. Kaplan–Meier curves of relapse-free survival in the training cohort **(A)** and the validation cohort **(B)** stratified by risk scores. Rank of risk scores and relapse status for each patient in the training cohort **(C)** and the validation cohort **(D)**. Heatmaps show the eight prognostic genes in the training cohort **(C)** and the validation cohort **(D)** between the high- and low-risk groups.

Based on the results of time-dependent ROC curve analysis, −17.519 and −16.832 were chosen as the optimal cut-off value for the training and validation cohorts, respectively. All patients were classified into either a high-risk group or a low-risk group according to their cutoffs. Patients with a high-risk score had a shorter RFS in both the training cohort (*P* < 0.001, Figure 3A) and validation cohorts (*P* = 0.021, Figure 3B). The distribution relapse status and relapse time for each patients in training (Figure 3C) and validation cohort (Figure 3D) were plotted with a division line by risk score cutoffs, which showed more relapse events in high-risk patients.

**Figure 4:**
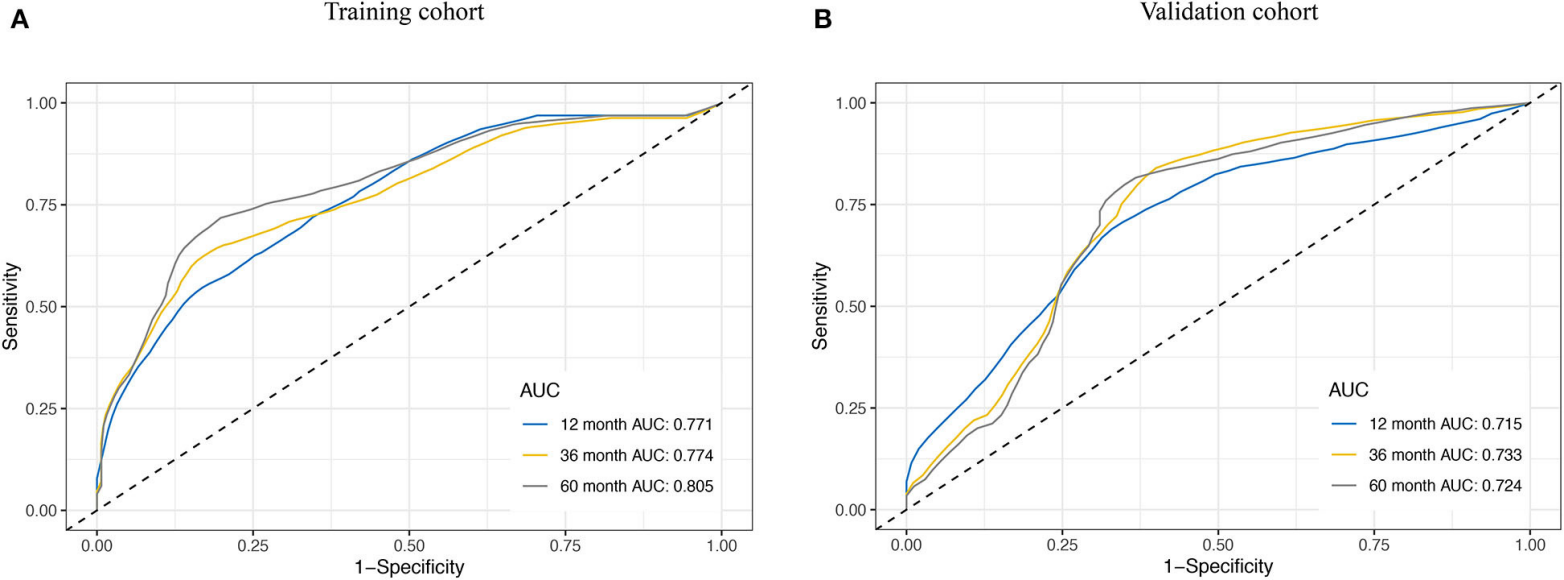
Receiver operating characteristic (ROC) curve analysis for the survival prediction model. The time-dependent ROC curves for relapse-free survival (RFS) were plotted at 1, 3, and 5 years to evaluate the prognostic performance of the eight-RNA risk score model in the training cohort **(A)** and the validation cohort **(B)**.

We assessed the prognostic accuracy of the risk score model with time-dependent ROC curve analysis at 1, 3, and 5 years in the training cohort and validation cohort (Figure 4). The AUC values of the eight-gene-based risk score model at 1, 3, and 5 years were 0.771, 0.774, and 0.805 in the training cohort, respectively. The AUC values of the risk score model at 1, 3, and 5 years were 0.715, 0.733, and 0.724 in the validation cohort, respectively.

As validation cohort had not enough clinical information, we only performed univariate and multivariate Cox regression analyses in TCGA-TGCT cohort to test the prognostic value of risk score. Univariate Cox regression revealed that serum markers stage, histological type, and risk score were prognostic factors for relapse in stage I TGCTs. Additionally, these significant prognostic factors were then investigated in multivariate Cox-regression analyses, and only risk score and histological type were the independent risk factors of TGCT relapse (Table 3). In different subgroups, the prognostic model also showed good performance in stratification (Table 4).

**Table 3:** Univariate and multivariate Cox regression analyses of risk factors associated with Relapse free Survival.

**Variables**	**Univariate analysis**		**Multivariate analysis**	
	**HR (95%CI)**	***P*-Value**	**HR (95%CI)**	***P*-Value**
Age	0.982 (0.942–1.023)	0.384		
**Race**				
Other	Ref			
White	1.432 (0.338–6.075)	0.626		
**Histological type**				
Seminoma	Ref		Ref	
Non-seminoma	2.404 (1.138–5.082)	0.022	2.902 (1.256–6.708)	0.013
**Lymphovascular invasion**				
No	Ref		Ref	
Yes	2.069 (0.996–4.298)	0.051	1.723 (0.774–3.834)	0.182
**Tumor (T) classification**				
Other	Ref			
T1	0.765 (0.36–1.625)	0.486		
**Serum markers (S) classification**				
Other	Ref		Ref	
S0	0.426 (0.182–0.998)	0.050	0.666 (0.255–1.738)	0.406
**Postoperative treatment**				
None	Ref			
Yes	0.554 (0.245–1.254)	0.157		
**Risk group**				
High risk	Ref		Ref	
Low risk	0.129 (0.059–0.284)	<0.001	0.093 (0.039–0.221)	<0.001

**Table 4:** Validation of the robustness of the prognostic value in in different subgroups.

**Variable**	**Count**	**Percent**	**HR**	**95% CI**	***p***
All	96	100.0	0.129	0.059–0.284	<0.001
**Race**					
White	87	90.6	0.154	0.068–0.346	<0.001
Other	9	9.4	0.000	0–Inf	1.000
**Histological type**					
Seminoma	60	62.5	0.076	0.02–0.282	<0.001
Non-seminoma	36	37.5	0.153	0.052–0.452	0.001
**Lymphovascular invasion**					
YES	34	35.4	0.208	0.072–0.603	0.004
NO	62	64.6	0.082	0.025–0.270	<0.001
**Tumor (T) classification**					
T1	64	66.7	0.098	0.036–0.266	<0.001
Other	32	33.3	0.151	0.043–0.533	0.003
**Serum markers (S) classification**					
S0	38	39.6	0.216	0.044–1.074	0.061
Other	58	60.4	0.098	0.036–0.266	<0.001
**Postoperative treatment**					
None	61	63.5	0.156	0.059–0.412	<0.001
Yes	35	36.5	0.113	0.027–0.478	0.003

### Functional Enrichment Analysis Between High- and Low-Risk Patients

**Figure 5:**
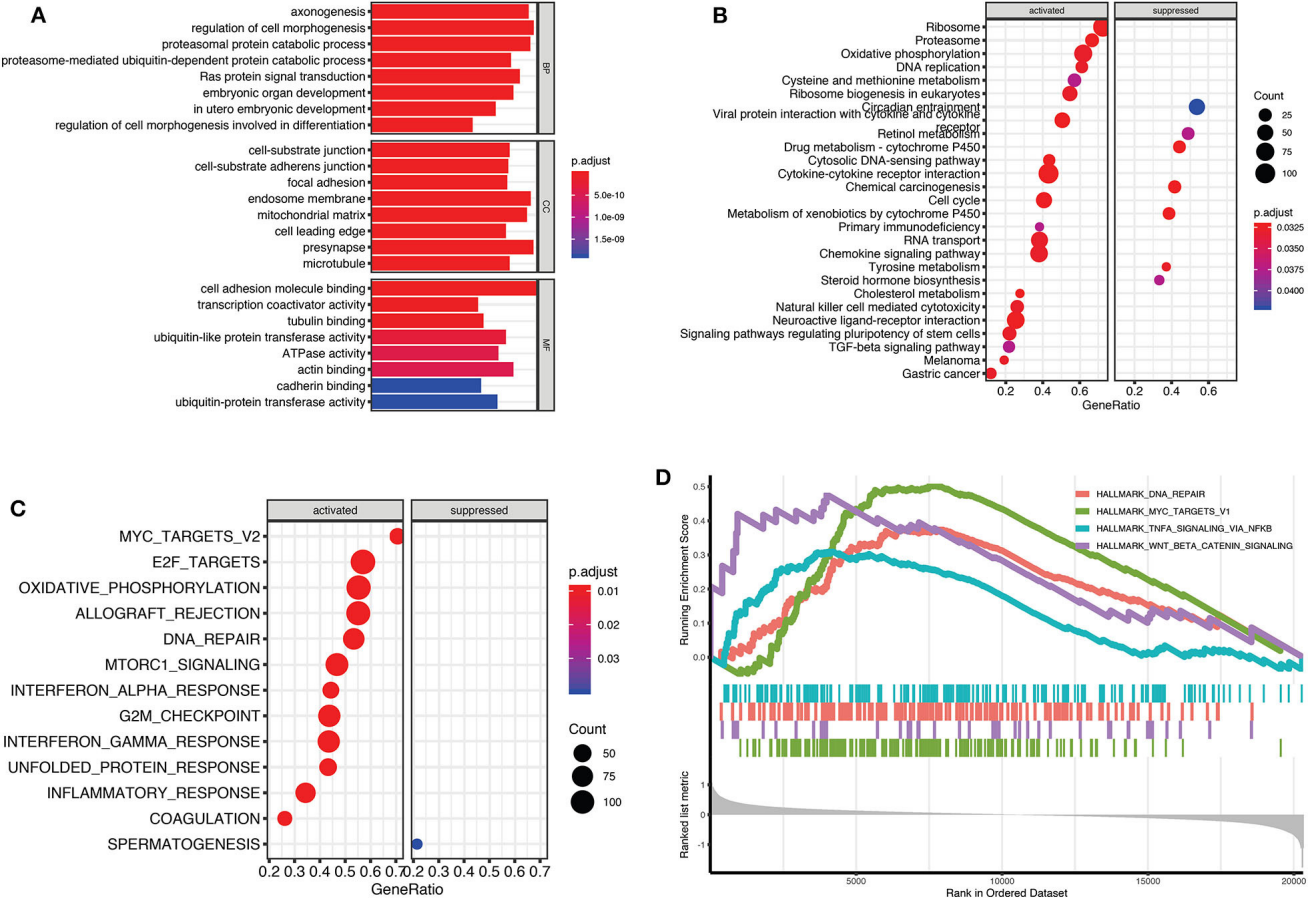
Gene Set Enrichment Analysis (GSEA) for Gene Ontology (GO) and Kyoto Encyclopedia of Genes and Genomes (KEGG) enrichment between high-risk and low-risk groups. **(A)** The top terms of molecular functions (MF), biological processes (BP), and cellular components (CC) in GO enrichment analysis. **(B)** GSEA-based KEGG analysis revealed the 27 significantly enriched pathways. **(C)** Activated and suppressed enrichment hallmarks terms in high-risk patients. **(D)** The visualization of some important enrichment hallmark terms.

To evaluate potential key molecules and pathways contributing to relapse, we performed GO analysis and GSEA between high-risk and low-risk patients. Top enriched GO terms in biological processes (BP), cellular components (CC), and molecular function (MF) are shown in Supplementary Tables 4–6, respectively. GO analysis revealed that axon genesis, regulation of cell morphogenesis, proteasomal protein catabolic process, and Ras protein signal transduction were the main terms involved in BP; cell–substrate junction, endosome membrane, and mitochondrial matrix were significantly enriched in CC; cell adhesion molecule binding, cytoskeletal protein binding, ATPase activity, and ubiquitin-like protein transferase activity were top enriched in MF (Figure 5A). Results of the KEGG pathway analysis showed that 27 pathways were enriched in high-risk TGCTs group (*P* < 0.05), among which the DNA replication, ribosome, cell cycle, and cytokine–cytokine receptor interaction signaling pathway may highly correlate with tumor relapse (Figure 5B, Supplementary Table 7). GSEA showed the significantly enriched hallmark terms associated with the relapse included DNA repair, MYC targets, and WNT β-catenin signaling (Figures 5C,D).

## Discussion

In the present study, we conducted a comprehensive genomic analysis of TGCTs and provided a prognostic risk model based on the expression of eight genes to predict relapse in patients with stage I TGCTs. Based on this model, the risk score was an independent prognostic factor for relapse in patients with stage I TGCTs.

Currently, TGCT patients have a 95% cure rate at first diagnosis and an expected 80% cure rate at the metastatic stage (4). However, the optimal management strategy for stage I TGCTs is one of the most controversial aspects of postoperative care (1, 5, 30, 31). Relapses are observed among 20–28% of TGCT patients who undergo surveillance after orchiectomy. Lack of satisfactory stratification that prevents overtreatment in low-risk patients and selection of patients with a high risk of relapse are difficult in clinical practice.

Classic clinical characteristics such as tumor type, serum tumor markers [alpha-fetoprotein (AFP), human chorionic gonadotropin (HCG), and lactate dehydrogenase (LDH)] and TNM stage can act as risk factors for relapse, but these clinical characteristics have low accuracy and low sensitivity for predicting relapse and are unable to meet the needs of clinical guidance. Terbuch et al. reported that serum miR-371a-3p is increased during TGCT recurrence in patients (32). The serum miRNA marker displays a diagnostic value for recurrence; however, it cannot be used for risk stratification in TGCT patients.

Gilbert et al. reported that CXCL12 expression can act as a prognostic index for stage I non-seminoma (33). Nevertheless, a meta-analysis summarized that no single risk factor could predict relapse in stage I seminoma (15). Korkola et al. identified an eight-gene model that can predict overall survival in patients with TGCTs in 2009, but without validation of RFS (34). Lewin et al. also reported a discriminatory gene expression profile between relapsed and non-relapsed cases on the basis of 10- and 30-gene signatures (35). However, the prediction score by Lewin et al. was limited to a cohort with single histology and lacked validation in large external sets. Our eight-gene-based risk score model had the ability to identify patients with a high risk of relapse and may suggest a risk-adapted adjuvant approach for TGCTs. The predictability was validated in an independent external cohort.

In this prognostic signature, seven had a protective role in TGCT relapse, and one was a risk factor for relapse in TGCT patients. By searching previous publications of those genes, results showed that all were newly reported associated with the prognosis of TGCTs. Some genes also play important biological roles in cancer development and progression. For example, SRARP (Steroid Receptor Associated And Regulated Protein) is a protein coding gene, which could function as a tumor suppressors and predict clinical outcome in malignancies (36). PLEKHS1 (pleckstrin homology domain containing S1) promoter mutations are a poor prognosis genetic marker for thyroid cancer (37).

GO analysis indicated that cell adhesion, cytoskeleton, Ras protein signal transduction, and autophagy most likely contribute to the relapse of TGCTs. From the results of GSEA-based KEGG pathway analysis, cell cycle, DNA replication, and TGF-beta signaling pathway were famous pathways of tumorigenesis; these pathways may be associated with metastatic behavior (38). Activated hallmark terms, such as MYC targets and WNT β-catenin signaling, also indicated the possible mechanisms of relapse in high-risk patients. For example, the activity of the MYC oncogene regulates tumor metastasis through specific effects on cancer cell invasion and migration (39). Wnt/β-catenin signaling could promote tumor metastasis via maintain epithelial-to-mesenchymal transition (EMT) or cancer stemness (40, 41). These pathways and detailed mechanisms that affect the relapse of TGCT require further investigation.

To the best of our knowledge, this is the first validated prognostic signature for stage I TGCT, and the present study included a large number of normal testis tissues and TGCT samples. The previous similar prognostic model study conducted by Korkola was based on microarrays and a small number of normal tissues (34). Our results provide novel biomarker candidates for TGCT studies and potential targets for treating TGCTs.

There are some limitations in the present study. The constructed risk score system was based on expression results, without consideration of the mutations, methylation status, or other genetic events that may be more important drivers of TGCTs. Further research on biological processes is still required to better understand the biology of TGCTs.

## Conclusions

In the present study, we developed a novel eight-gene-based risk model for predicting the RFS of stage I TGCT patients. The relapse risk prediction model provides an approach to individualize treatment decisions for stage I TGCT patients.

## Data Availability Statement

The datasets generated for this study can be found in the TCGA and GSE99420.

## Author Contributions

J-GZ, JY, S-HJ, and HM conceived, designed, or planned the study. JY and J-GZ analyzed the data. T-YZ and S-HJ acquired the data. J-GZ, S-HJ, LS, and HM helped interpret the results. J-GZ and JY provided study materials or patient data. J-GZ, JY, SX, S-HJ, UG, and HM drafted the manuscript. All authors revised and reviewed this work and gave their final approval of the submitted manuscript.

## Conflict of Interest

The authors declare that the research was conducted in the absence of any commercial or financial relationships that could be construed as a potential conflict of interest.

## Abbreviations

TCGA: The Cancer Genome Atlas
GTEx: Genotype-Tissue Expression Project
GEO: Gene Expression Omnibus
TGCTs: testicular germ cell tumors
FDR: false discovery rate
ROC: receiver operating characteristic
AUC: area under the ROC curve
GO: Gene Ontology
KEGG: Kyoto Encyclopedia of Genes and Genomes
MF: molecular functions
BP: biological processes
CC: cellular components
TPM: transcripts per kilobase million
CI: confidence interval
HR: hazard ratio
DEGs: differentially expressed genes
RFS: relapse-free survival
PCA: principal component analysis
LASSO: least absolute shrinkage and selection operator.
